# Efficacy and safety of hydro-mechanical defragmentation in intermediate- and high-risk pulmonary embolism

**DOI:** 10.1186/s43044-021-00204-2

**Published:** 2021-09-25

**Authors:** Ayman K. M. Hassan, Heba Ahmed, Yousef Ahmed, Abd-Elazim Abo Elfadl, Amany Omar

**Affiliations:** 1 Cardiology Department, Assiut University, P.Box: 71526, Asyut, Egypt; 2 Chest Department, Assiut University, Asyut, Egypt

**Keywords:** Catheter-directed therapy, Pulmonary embolism, Hydro-mechanical defragmentation, Systemic thrombolysis, High risk

## Abstract

**Background:**

Pulmonary embolism (PE) is the third most common acute cardiovascular syndrome. Percutaneous catheter directed hydro-mechanical defragmentation (HMD) is one of the recommended treatment options for PE in patients with contraindications to thrombolytic therapy or failed systemic thrombolysis (ST). We aimed to identify the safety and outcomes of catheter directed HMD in patients with high-risk PE. This nonrandomized controlled trial enrolled all patients with confirmed diagnoses of high- and intermediate-high-risk PE from October 2019 till January 2021. Fifty patients were included and divided into two groups by the PE response team according to the presence or absence of a contraindication for ST. Group B (ST) consists of 25 patients and group A (HMD) of 25 patients who cannot receive ST.

**Results:**

The two groups were comparable regarding baseline clinical characteristics with mean age 51 ± 13 years. In group A, systolic blood pressure (BP) and oxygen saturation increased after 24 h (*p* = 0.002) and 48 h (*p* < 0.001) compared to pre-HMD procedure. Mean pulmonary artery systolic pressure (PASP) and respiratory rate (RR) decreased after 48 h and at 30 days (*p* < 0.001) compared to pre-HMD procedure. The increase in systolic BP and oxygen saturation were significantly higher in HMD group compared with ST group after 48 h and at 30 days (*p* < 0.007). The decrease in PASP and RR was significantly higher in HMD group compared to ST group after 48 h and at 30 days (*p* < 0.001). Mortality rate at 30 days was 20% in HMD group compared to 32% in ST group.

**Conclusions:**

Catheter directed HMD for high-risk and intermediate-high-risk PE is safe and effective with acceptable mortality

*Trial registration* Clinical trial ID: NCT04099186.

## Background

High-risk PE is an immediately life-threatening situation that requires an emergency diagnostic and therapeutic strategy [1]. Hemodynamic instability and right ventricular failure indicate a high risk of early mortality. However, hemodynamic stability does not exclude beginning of right ventricular dysfunction that may also be progressing, and thus a high PE-related risk as mentioned in recent guidelines as intermediate-high-risk PE group [2].

Systemic intravenous thrombolysis (ST) is recommended by all guidelines for high-risk PE but remains disputable for intermediate-high-risk PE [3]. ST and embolectomy have the potential to reduce right ventricular pressure overload in high-risk [4] and intermediate-high-risk PE [5] and decrease mortality by reversing pulmonary arterial obstruction and RV failure. However, this benefit was counterbalanced by an early fivefold increased risk of major bleeding and a tenfold increased risk of hemorrhagic stroke [6].

Bleeding complications, absolute and relative contraindications for ST have generated interest in alternative therapies with lower bleeding risk.

Catheter-directed therapies (CDT) include different types of catheters for mechanical fragmentation, thrombus aspiration, or more commonly a pharmaco-mechanical approach combining mechanical or ultrasound fragmentation of the thrombus with in situ reduced-dose thrombolysis [1]. The efficacy of thrombus fragmentation or aspiration techniques only with no lytic agents, for patients with absolute contraindications to thrombolysis, remains controversial [7, 8]. Thrombus fragmentation using rotating pigtail catheter has been widely reported and used [9]; it is easily available and comes at a low cost. As it leads to peripheral clot embolization, parallel aspiration thrombectomy may be required. Unless the embolus is being fragmented to allow a greater embolic surface area for the lytic drug to work on, thrombolytic infusion into the pulmonary artery proximal to the embolus will have no benefit compared to systemic delivery as it will wash into nonoccluded vessels rapidly [10, 11].

The procedural success rates in CDT were defined as hemodynamic stabilization, correction of hypoxia, and survival to hospital discharge with an overall procedural success reported in these studies have reached 87% [12].

Recent guidelines upgraded the CDT to class IIA recommendation stating that it should be considered for patients with high-risk PE, in whom thrombolysis is contraindicated or has failed [1].

Hydro-mechanical defragmentation (HMD) is one of the CDT modalities for high-risk PE patients, in which rapid pigtail rotation is combined with heparinized saline injection for thrombus fragmentation. In this study, we aimed to identify HMD safety and outcomes in high-risk and intermediate-high-risk PE patients compared to the conventional ST approach.

## Methods

### Study design and setting

This is an interventional prospective case–control study. We evaluated all adult patients who were presented to the emergency department with clinical manifestations of acute pulmonary embolism (PE) as a part of pulmonary embolism response team (PERT). PERT ask for an emergency computed tomographic pulmonary angiography (CTPA) and laboratory routine tests including troponin level to confirm diagnosis of PE and risk stratify patients in to low-, intermediate-low-, intermediate-high- and high-risk PE. To be included in the study, patient with confirmed PE by CTPA should be presented within first 24 h of onset of symptoms with sings of hemodynamic instability to be diagnosed as high-risk PE. Or patients with combined PE severity index (PESI) class III/IV, RV dysfunction on echocardiography (ECHO) and elevated cardiac troponin level without evidence of hemodynamic instability to be diagnosed as intermediate-high-risk PE as clarified in 2019 ESC guidelines [1]. We excluded patients with low-risk and intermediate-low-risk pulmonary embolism and those who refused to be included in the study.

The study was approved by the Assiut University institutional review board (IRB No. = 17200222) and complies with the Declaration of Helsinki. Privacy and confidentiality of all the data were assured. The aim of the study was explained to each participant before the procedure. Informed written consent was obtained from each participant in the study. The research project is registered on ClinicalTrial.gov (registration No. = NCT04099186); registered 23 September 2019; https://www.clinicaltrials.gov/ct2/show/NCT04099186.

### Study participants

**Fig. 1:**
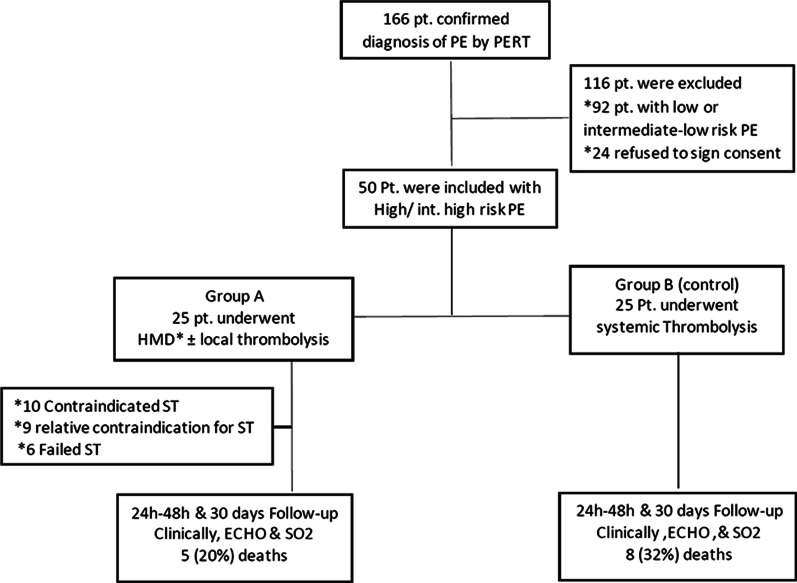
Flowchart of the study population. All 166 patients were first diagnosed by PERT who decide the treatment strategy. HMD: hydro-mechanical defragmentation, ST = systemic thrombolysis, PERT = pulmonary embolism response team, PE: pulmonary embolism, pt. = patients, ECHO = echocardiography, SO2 = oxygen saturation

Between October 1, 2019, and January 31, 2021, the PERT confirmed the diagnosis of PE in 166 patients. One hundred and sixteen patients were excluded (92 patients with low- or intermediate-low-risk PE and remaining 24 patients refused to be included in the study). We recruited 50 patients with confirmed acute high- or intermediate-high-risk PE. Patients were then classified into two groups based on the following protocol. Once patient accepted in the study, PERT decide if there is a contraindication or not for systemic thrombolysis. If no contraindication for systemic thrombolysis, patients were included in group B and received systemic thrombolysis and reassessed after 24–48 h for the response. But if there was any contraindication for or failed management with ST, patients were included in group A and transferred to cardiology catheterization laboratory for hydro-mechanical defragmentation (HMD) of the thrombus and continue local heparin infusion for 48 h. Local injection of ST was given after HMD, if relative contraindication for SK was diagnosed by the PERT. Absolute ST contraindications include history of hemorrhagic stroke or stroke of unknown origin, ischemic stroke in previous 6 months, central nervous system neoplasm, major trauma, surgery, or head injury in previous 3 weeks, bleeding diathesis, and active bleeding. Relative ST contraindications include transient ischemic attack in previous 6 months, oral anticoagulation, pregnancy or first post-partum week, non-compressible puncture sites, traumatic resuscitation, refractory hypertension (systolic BP > 180 mmHg), advanced liver disease, infective endocarditis, and active peptic ulcer [1]. The flowchart of the study is shown in Fig. 1.

### Study variables and data measurements

All patients were subjected to full medical history taking and clinical examination, including BP measurement, CTPA, oxygen saturation measurement, and routine laboratory blood sample analysis, including troponin measurement at presentation, arterial blood gases (ABG), and continuous monitoring for clinical and hemodynamic status in ICU. Patients were divided to two groups, 25 patients in each group. Group A underwent HMD fragmentation of pulmonary embolism with or without local thrombolysis. Group B took systemic thrombolytic.

### Resting transthoracic 2D echocardiography (ECHO)

Resting transthoracic 2D echocardiography (ECHO) was performed on all patients at presentation, after 48 h, and after 30 days by the same machine (VIVID S5 instrument, GE Medical Systems, Horten, Norway) for 2D data. ECHO was performed based on the European Association of Echocardiography (EAE) [13] recommendations and the American Society of Echocardiography (ASE) [14]. Pulmonary artery systolic pressure and signs of RV dysfunction as dilated right side, positive McConnell sign, and 60/60 sign were obtained. Pulmonary artery systolic pressure (PASP) was calculated using the Bernoulli equation using the tricuspid regurge (TR) velocity 4(VTR)2 + RAP (VTR = TR maximal velocity, RAP = the estimated right atrial pressure). The calculation is not valid if there is severe (free) TR, tricuspid stenosis or tricuspid prosthetic valve, RV systolic dysfunction (e.g., RV infarct), and if there is no TR, it does not mean that there is no pulmonary hypertension.

### Hydro-mechanical defragmentation (HMD) technique

The interventional procedure was done in cardiac catheterization laboratory (Phillips catheter lab). The patient received local anesthesia, and then a (6)F sheath was introduced in the femoral vein for procedure.

Then, a (6)F multipurpose catheter was advanced over a 0.35 j tip guide wire under fluoroscopic guidance and used to measure right heart and pulmonary artery pressures.

**Fig. 2:**
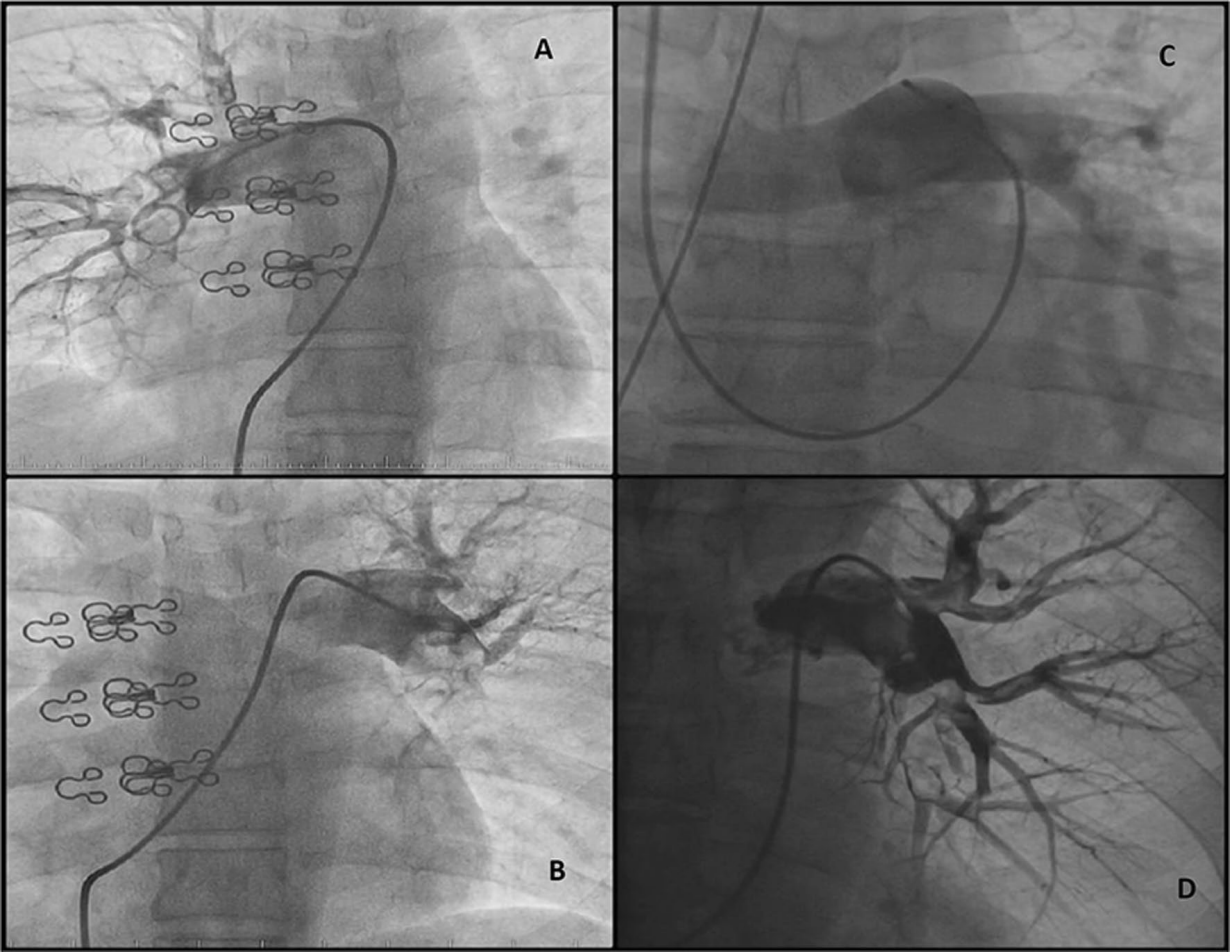
Pigtail catheter in different patients’ pulmonary artery. **a** Pigtail cath. In right main pulmonary artery (PA) with thrombus seen at tip of catheter using 20-ml contract injection. **b** The pigtail cath. in left main PA showing a thrombus at tip of cath. before fragmentation. Clips of patient spinal surgery is seen in both frames. **c** Pigtail cath. in main PA before bifurcation in another patient using right jugular vein for cath. insertion. **d** Pigtail cath.in left PA after successful fragmentation with residual thrombus in distal LPA but opened distal vessels

Mechanical fragmentation was done using a 6 F pigtail catheter inserted inside the thrombus guided by the CTPA images. The catheter was quickly spun in a rotatory movement manually to fragment the central thrombus and establish initial flow into pulmonary artery followed by rapid intrapulmonary injection of 100–200 ml heparinized saline by an injector to help in hydro-mechanical defragmentation of the thrombus. Final angiography of the pulmonary circulation was done, if not risky, to ensure establishing flow in distal arteries (Fig. 2).

Then, we leave the catheter in situ and continue intrapulmonary heparin injection for 24–48 h. Then, reassess the PA pressure by catheter and finally remove the pigtail.

Timing for HMD: Procedure was done as early as possible after initial diagnosis within 1st 12–24 h.

Fifteen patients in HMD group, with relative contraindication to ST, had an injection of ST via pigtail for 24-h intrapulmonary. After ensuring initial flow by HMD, initial bolus dose of streptokinase (250,000 IU) was given over 1 h followed by continuous infusion for 24 h at a rate of (100,000 IU/h).

#### Systemic thrombolysis

Patients with no contraindication for ST were included in group B and admitted in ICU and received ST. Initial bolus dose of ST was given over 1 h followed by continuous infusion for 24 h [1]. After HMD or systemic thrombolysis, patient is maintained on systemic heparin infusion at initial dose of 18 IU/kg and adjusted to keep activated partial thromboplastin time (APTT) to 1.5 or more times the control value [15], then on discharge, oral anticoagulant (VKA or NOAC) is prescribed for ⩾3 months [1].

#### Study outcomes and clinical follow-up

All patients were followed up for 24, 48 h and at 30 days after discharge. Mean duration for hospitalization was 9 days. Primary efficacy outcome was hemodynamic stabilization of the patients including elevation of systolic BP, reduction in respiratory and heart rates and improvement in oxygen saturation at follow-up. Shock index was measured (heart rate divided by systolic blood pressure, with a normal range of 0.5–0.7 in healthy adults) [16]. Secondary efficacy outcome was reduced in PASP measured invasively at 48 h, and by Echo at 48 h and 30th day. The safety outcomes include mortality rate and bleeding complication during hospitalization and at 30-day follow-up. Bleeding is classified to major bleeding and minor bleeding according to Steeple bleeding criteria [17].

### Statistical analysis

Data were verified and coded by the researcher, and then analyzed using IBM-SPSS 24.0 (IBM-SPSS Inc., Chicago, IL, USA). Descriptive statistics, means, standard deviations (SD), median and range, were calculated. Test of significances, Chi-square/Fisher’s Exact/Monte Carlo exact test, was calculated to compare the frequencies among groups. Independent t test analysis was carried out to compare the means of dichotomous data. One-way ANOVA was used to test the mean differences of the data that follow normal distribution; post hoc test was calculated using Bonferroni corrections for pairwise comparisons between the two study groups. A significant p value was considered when it is < 0.05.

## Results

The current study included 50 patients with high-risk and intermediate-high-risk PE. Patients were divided to two groups.

### Demographic and descriptive data are shown in Table 1

**Table 1:** Clinical, demographic and investigational characteristics of the study groups

Characteristics	Group A (HMD)	Group B (ST)	*p* value
	No. 25	No. 25	
Age (range) year	49.6 ± 13.13 (26–68)	42.92 ± 12.3 (28–80)	0.070
Female gender (%)	13 (52%)	17 (68%)	0.248
Risk factor for pulmonary embolism			
Major surgery	6 (24%)	10 (40%)	0.363
Fracture	6 (24%)	3 (12%)	0.462
Cancer	3 (12%)	1 (4%)	0.602
Paraplegia	1 (4%)	0 (0%)	1.000
Contraceptive pills	3 (12%)	4 (16%)	1.000
Stroke	0 (0%)	2 (8%)	0.470
Unknown	6 (24%)	5 (20%)	1.000
CTPA results on admission (%)			
Bilateral main PE	21 (84%)	17 (68%)	0.069
Saddle shaped PE	4 (16%)	2 (8%)	
Right main PE	0	4 (16%)	
Left main PE	0	2 (8%)	
Echocardiography (%)			0.97
Dilated right side	25 (100%)	25 (100%)	0.069
60/60 sign	25 (100%)	25 (100%)	
McConnell sign	25 (100%)	25 (100%)	
Type of pulmonary embolism(%)			
High-risk PE	17 (68%)	17 (68%)	0.989
Intermediate-high-risk PE	8 (32%)	8 (32%)	
Procedure done (%)			
Fragmentation only	10 (40%)	0	
Fragmentation and local thrombolysis	9 (36%)	0	
Fragmentation and local thrombolysis after failure of ST	6 (24%)	0	

Data are presented as mean ± standard deviation or number and percent. HMD = hydro-mechanical defragmentation; ST = systemic thrombolysis; PE = pulmonary embolism; CTPA = computed tomography pulmonary angiography; yr. = years

*Statistically significant difference (*p* < 0.05); **Highly statistically significant difference (*p* < 0.01)

The mean age in group A was 49.6 ± 13.13 years with 52% females, and 42.92 ± 12.3 in group B with 68% female (Table 1).

Regarding risk factors, 32% of patients underwent a recent major surgery, 18% had a recent fracture, 8% were diagnosed with a malignant tumor, 14% were on oral contraceptive pills, 4% had a recent stroke, while 22% had no risk factors (Table 1). All patients in both groups had raised troponin level.

No significant differences were observed between invasive PASP assessment compared to noninvasive assessment by Echo (57.8 ± 7 vs 59.6 ± 8, *p* = 0.09), respectively.

### Results for group A (HMD)

*Systolic blood pressure* significantly increased from 87.2 ± 17.68 before procedure to 98 ± 16.24 after 24 h and 108.7 ± 10.14 after 48 h (*p* < 0.001).

*Oxygen saturation* increased from 88.44 ± 4.42 before procedure to 91.71 ± 3.09 after 24 h and 92.56 ± 4.77 after 48 h (*p* < 0.001).

*Respiratory rate *decreased from 37.68 ± 4.29 before procedure to 30.79 ± 3.91 after 24 h and 26.83 ± 5.08 after 48 h (*p* < 0.001).

*Pulmonary artery* systolic pressure decreased from 60.63 ± 9.22 before procedure to 45.12 ± 12.97 after 48 h and 36 ± 14.07 after 30 days (*p* < 0.001). *Reduction of PASP took longer time to be manifested significantly mostly due to neuro-hormonal activation and increased pulmonary vascular resistance.*

### Results comparing group A (HMD) versus group B (ST)

All efficacy parameters of the HMD technique showed significant improvement compared to control group as shown in Tables 2 and 3.

**Table 2:** Efficacy parameters of the study groups comparing admission and follow-up results

	Group A (HMD)	Group B (ST)	*p* value
	No. 25	No. 25	
Systolic blood pressure			
Before procedure	87.2 ± 17.68	85.2 ± 7.14	0.602
After 24 h	98.75 ± 16.24	89.2 ± 12.22	0.024
After 48 h	108.7 ± 10.14	94.09 ± 11.82	< 0.001**
After 30 day	119 ± 6.41	109.47 ± 18.1	0.033*
Diastolic blood pressure			
Before procedure	53.2 ± 16	52.8 ± 7.92	0.911
After 24 h	63.75 ± 9.24	55.6 ± 8.7	0.003**
After 48 h	68.7 ± 7.57	59.55 ± 7.22	< 0.001**
After 30 day	76 ± 5.03	70 ± 12.47	0.054
Pulmonary artery systolic pressure			
Before procedure	59.68 ± 8.35	52.2 ± 6.63	< 0.001**
After 48 h	46.1 ± 12.18	48.67 ± 6.4	0.464
After 30 day	36.5 ± 10.35	42.24 ± 6.6	0.065
Respiratory rate			
Before procedure	37.68 ± 4.29	36.8 ± 6.79	0.586
After 24 h	30.79 ± 3.91	32.28 ± 5.06	0.257
After 48 h	26.83 ± 5.08	30.73 ± 5.1	0.013*
After 30 day	19.4 ± 1.73	21.47 ± 2.29	0.004**
Oxygen saturation			
Before procedure	88.44 ± 3.94	92.28 ± 2.56	< 0.001**
After 24 h	91.71 ± 3.09	92 ± 3.11	0.743
After 48 h	92.65 ± 4.77	92.82 ± 3.36	0.894
After 30 day	95.4 ± 1.14	96.47 ± 1.62	0.025*
Heart rate			
Before procedure	130.8 ± 10.38	124.8 ± 8.23	0.028*
After 24 h	117.5 ± 8.97	117.6 ± 8.79	0.969
After 48 h	110 ± 7.98	110.91 ± 6.84	0.684
After 30 day	95.5 ± 5.1	93.44 ± 11.35	0.469
Shock index (HR/SBP)			
Before procedure	1.54 ± 0.4	1.44 ± 0.18	0.276
After 24 h	1.21 ± 0.28	1.32 ± 0.26	0.142
After 48 h	1 ± 0.14	1.2 ± 0.24	<0.001**
After 30 day	0.78 ± 0.1	0.77 ± 0.1	0.940

Data are presented as mean ± standard deviation. HMD = hydro-mechanical defragmentation; ST = systemic thrombolysis; PE = pulmonary embolism. * Statistically significant difference (*p* < 0.05); ** Highly statistically significant difference (*p* < 0.01)

**Table 3:** Rate of change in efficacy parameters of the study groups subtracting the previous value from the follow-up results

The rate of change^a^	Group A (HMD)	Group B (ST)	*p* value
	No. 25	No. 25	
Increase in SBP			
After 24 h	12.08 ± 9.32	9.6 ± 4.55	0.239
After 48 h	20.87 ± 13.11	12.27 ± 5.28	0.007**
After 30 day	32 ± 16.73	26.84 ± 9.46	0.247
Increase in DBP			
After 24 h	11.25 ± 11.54	6 ± 6.45	0.054
After 48 h	15.22 ± 12.75	8.18 ± 5.88	0.023*
After 30 day	23 ± 16.25	18.16 ± 9.01	0.261
Decrease in PASP			
After 48 h	13.89 ± 8.05	1.53 ± 2.29	< 0.001**
After 30 day	20.41 ± 7.13	8.18 ± 3.32	< 0.001**
Decrease in respiratory rate			
After 24 h	6.88 ± 2.79	5.24 ± 4.7	0.147
After 48 h	10.71 ± 5	6.45 ± 5.47	0.008**
After 30 day	17.5 ± 4.37	14.24 ± 6.19	0.069
Increase in oxygen saturation			
After 24 h	3.58 ± 2.5	0.68 ± 1.31	< 0.001**
After 48 h	4.74 ± 2.8	1.18 ± 1.59	< 0.001**
After 30 day	6.35 ± 2.83	2.83 ± 1.2	< 0.001**
Decrease in heart rate			
After 24 h	13.48 ± 9.82	9.2 ± 6.4	0.078
After 48 h	21.82 ± 10.53	13.64 ± 9.02	0.008**
After 30 day	35.5 ± 10.99	30.44 ± 9.88	0.146

Data are presented as mean ± standard deviation. HMD = hydro-mechanical defragmentation; ST = systemic thrombolysis; PE = pulmonary embolism; SBP = systolic blood pressure; DBP = diastolic blood pressure; PASP = pulmonary artery systolic pressure

*Statistically significant difference (*p* < 0.05); **Highly statistically significant difference (*p* < 0.01). ^a^The change is measured by subtracting the previous value to calculate the rate of change in each efficacy parameter

**Fig. 3:**
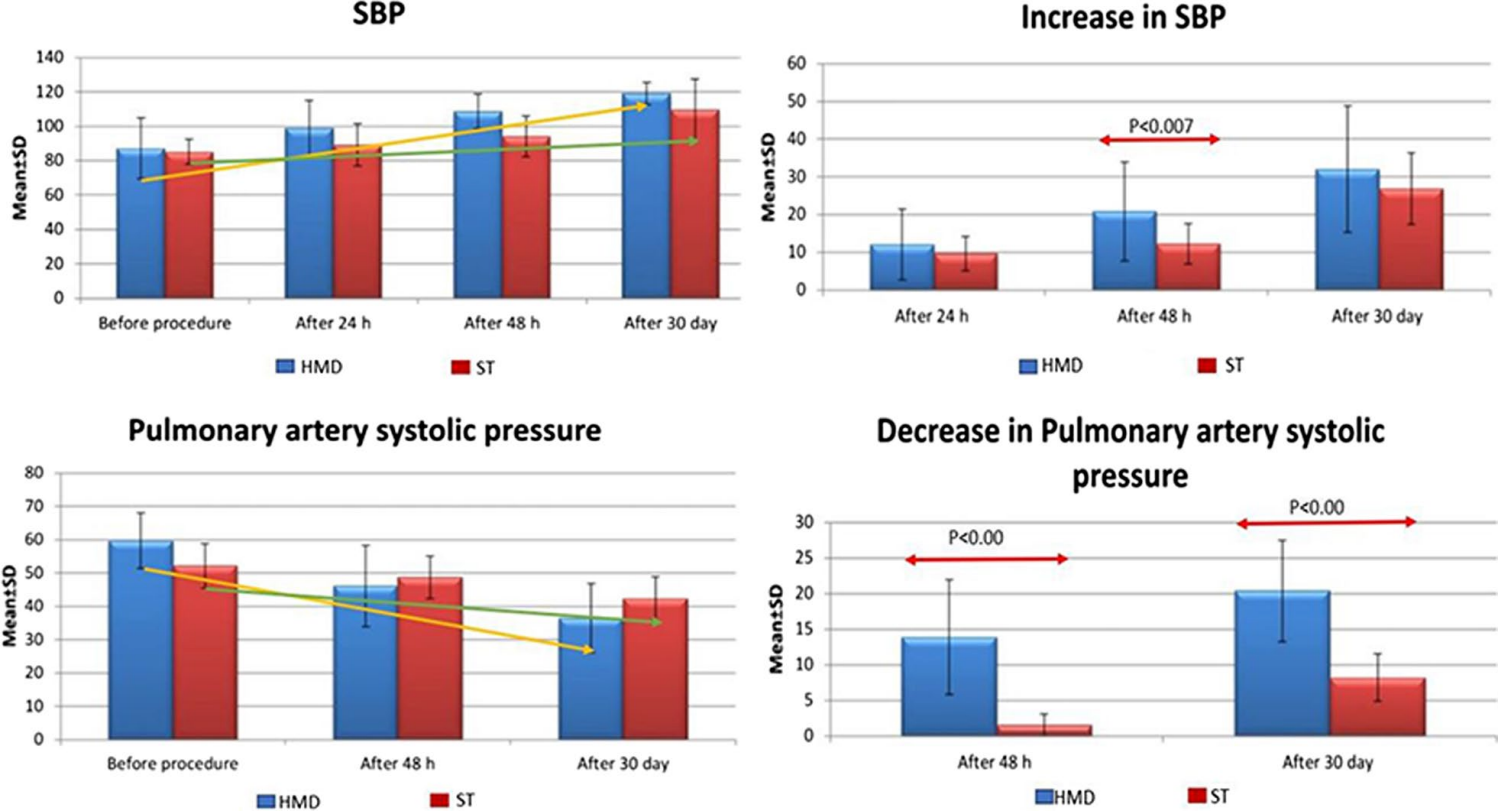
Rate of change in systolic blood pressure and pulmonary artery systolic pressure between study groups at admission and during follow-up

*The increase in systolic blood pressure was *significantly higher in HMD group compared to ST group after 48 h and at 30 days (*p* < 0.007). (Fig. 3).

**Fig. 4:**
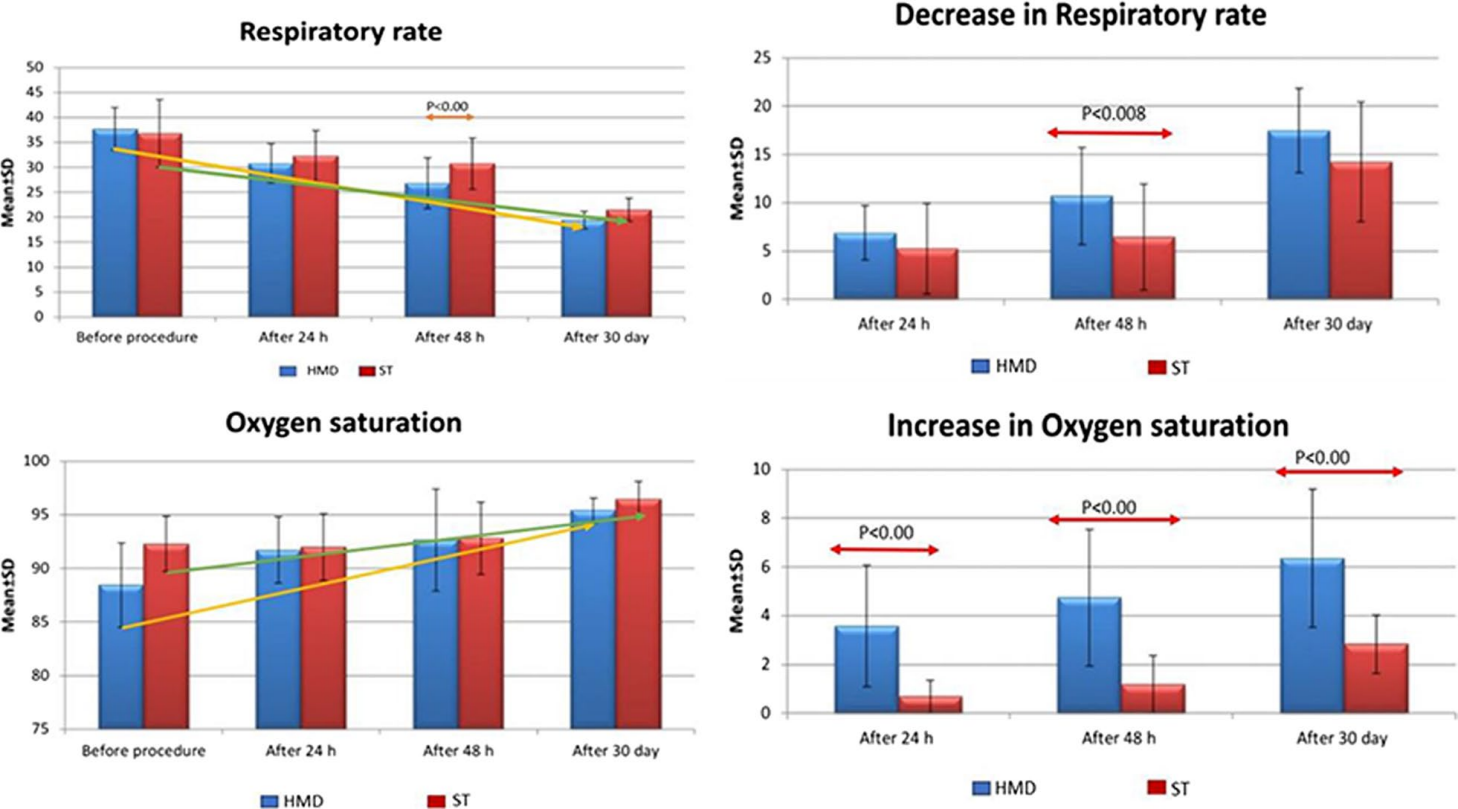
Rate of change in respiratory rate and oxygen saturation between study groups at admission and during follow-up

*The decrease in pulmonary artery* systolic pressure was significantly higher in HMD group compared to ST group after 48 h and after 30 days (*p* < 0.001) (Fig. 4).

*The decrease in respiratory rate *was significantly higher in HMD group compared to ST group after 48 h and after 30 days (*p* < 0.001).

*The increase in* oxygen saturation *was* significantly higher in HMD group compared to ST group after 24, 48 h and at 30 days (*p* < 0.007).

The HMD procedure was safe and effective especially when done within first 12 h after diagnosis.

### Safety outcomes are given in Table 4

**Table 4:** Safety outcomes measures of the study groups at discharge and during follow-up

Safety outcomes	Group A (HMD) No = 25	Group B (ST) No = 25	*p* value
Bleeding complications			0.401
Major bleeding	0	1 (4%)	
Minor bleeding	1 (4%)	3 (12%)	
In hospital mortality			0.333
Death	5 (20%)	8 (32%)	
Survival	20 (80%)	17 (68%)	
30-day mortality			0.333
Death	5 (20%)	8 (32%)	
Survival	20 (80%)	17 (68%)	

Data are presented as number and percent. HMD = hydro-mechanical defragmentation; ST = systemic thrombolysis; *Statistically significant difference (*p* < 0.05)

Mortality rate in HMD group was 20% (four patients died after doing HMD late after 12-18 h of diagnosis, one patient died from sepsis due to hospital acquired pneumonia) compared to 32% (seven patients died due to persistent shock not responding to inotropes and one patient died due to cerebral hemorrhage) in ST group.

No major bleeding occurred in group A compared to one case of major bleeding (4%) occurred in group B; minor bleeding occurred in 4% of group A compared to 12% in group B (Table 4).

## Discussion

HMD is a newly modified technique that improved the results of pigtail mechanical fragmentation in both high-risk and intermediate-high-risk PE patients. The main finding of our results is:

1. HMD is effective as it significantly improves systolic blood pressure, RR, oxygen saturation and PASP both in high-risk and intermediate-high-risk PE patients.
2. HMD is safe with non-significant trend toward lower mortality and bleeding risk when compared to conventional ST.

Catheter-directed therapy for acute PE is a bail out treatment option and its role is rapidly evolving. The current guidelines have accepted CDT as a treatment option for high-risk PE in patients with contraindications to thrombolysis, failed thrombolysis, or in cases with shock that is likely to cause death before systemic thrombolysis can take effect [1].

Different catheters and devices have been used to fragment large centrally located emboli by direct mechanical action [18]. The ideal catheter system for treatment of acute high-risk PE should be rapidly placed and well-steerable in all parts of the pulmonary artery system. Ease of handling is an important feature, saving time and avoiding complications in an emergency procedure. The pigtail catheter used in this study is universally available affordable and effective device. The pigtail rotation catheter concept was chosen under the consideration that the pigtail tip is the safest configuration for probing of the pulmonary arteries. The pigtail tip avoids perforation and allows easy manipulation into and within the pulmonary arteries [8].

The aim of therapy is to shatter the large embolus by rotatory movement and saline injection into multiple small fragments to obtain partial reperfusion. Fragmentation of centrally located, large pulmonary emboli may lead to a partial recanalization of a complete occlusion. In patients threatened by right ventricular failure, even a small hemodynamic improvement may be life-saving and enlarges the critical time frame for further recanalization by medical thrombolysis. This also gives a better thrombolytic action when local thrombolysis is added in cases without absolute contraindication for thrombolytic therapy, as this increase the exposure of fresh clot surfaces caused by fragmentation improving the thrombolytic action, as when there is complete occlusion of pulmonary artery by a thrombus, any infused drug will make only brief contact with embolus and move into the nonoccluded patent branches. After fragmentation, infused thrombolytics will have better contact with the distal embolus fragments [8].

We suggest that rapid reperfusion of pulmonary arteries with mechanical fragmentation pigtail catheter followed by intrapulmonary injection of thrombolytic over 24 h improves the outcomes compared to IV thrombolysis in patients presenting with high-risk or intermediate-high-risk pulmonary embolism.

In this study, we reported a success rate of 80% with low risk of bleeding in cases who were subjected to HMD; this agrees with a previous meta-analysis reporting 86.5% clinical success [7] and consistent with a recent study where 87.5% success rate was reported [19]. Our success rates also agrees with two recent trials showing a high success rate of CDT for PE with a low risk of significant bleeding [2, 20] despite the use of expensive ultrasound-assisted catheters in the **SEATTLE II** [2] and **ULTIMA** [20] trials.

For the catheter embolectomy procedure, Greenfield et al. reported a 30-day survival rate of 70% [21] and Timsit et al. reported an overall long-term survival rate of 72% [8].

We observed lower rate of bleeding (only one case with minimal bleeding with no major bleeding) in HMD group compared to ST group; this agrees with the **SEATTLE II** trial [2] who reported no hemorrhagic strokes and only one severe bleeding event and **ULTIMA** [20] trial where no bleeding events were reported.

Our results supports the concept of early CDT intervention, as four of the five failed cases were presented lately after 24 h of shock onset; this agrees with the **PERFECT** [19] trial.

We observed greater decrease in PASP in HMD group than ST group. Also the decrease in PASP was greater in cases who were subjected to HMD and local thrombolysis than in cases who were subjected to HMD alone. So we believe mechanical fragmentation is better to be followed by catheter-directed thrombolysis in cases without absolute contraindication to thrombolytic therapy to achieve the best result. This is consistent with a recent study (Bishav Mohan et al.) [22] who reported a 41% reduction in mean pulmonary artery pressure at 24 h, and also consistent with William et al. (2015) [23] and Navkaranbir et al. (2016) [24] reported significant improvement in PASP after CDT.

This also agrees with the **PERFECT** [19] trial who observed that low-dose CDT infusion improves pulmonary hypertension, RV strain, and hemodynamic parameters.

In our study, we observed a non-significant trend toward lower mortality rate in the HMD group compared to ST group mostly due to low power of our study. This agrees with Stephen D’Auria et al. [25] and Lukas Hobohm et al. [26] who reported lower mortality rate in PE patients who were subjected to CDT compared to patients who received ST.

In this current study, CDT was found to significantly improve oxygen saturation, blood pressure, and shock index. This agrees with Bishav Mohan et al., 2014 [22] who reported significant improvement in oxygen saturation, MBP, and shock index.

*Main limitations of our study were*: It is a nonrandomized study where bias in choosing management options cannot be ignored. However, the PERT included different specialists from different departments, and their diagnosis and clinical evaluation of the patients was independent. The COVID 19 pandemic reduced our HMD-intended group due to hospital regulation. Relatively small sample size resulted in non-significant difference in mortality rates, further studies are needed with larger number and better randomized. We cannot exclude delay in diagnosis as we included cases from different departments as vascular surgery and orthopedic surgery which prevent time-delay comparison between groups.

## Conclusions

HMD is a safe and effective modality for treatment of high-risk and intermediate-high-risk pulmonary embolism that can be used as a first line treatment or after failure of ST with improved overall clinical outcomes and non-significant trend toward lower mortality rate or bleeding.

## Data Availability

Data included excel sheets, and BP results are available on reasonable request.

## Abbreviations

ABG: Arterial blood gases
APTT: Activated partial thromboplastin time
CDT: Catheter-directed therapies
CTPA: Computed tomographic pulmonary angiography
ECHO: Echocardiography
HMD: Hydro-mechanical defragmentation
ST: Systemic intravenous thrombolysis
PE: Pulmonary embolism
PERT: Pulmonary embolism response team
PESI: PE severity index
